# Medullary Thyroid Carcinoma with Micronodular Lung Metastases: A Case Report with an Emphasis on the Imaging Findings

**DOI:** 10.1155/2010/616580

**Published:** 2010-05-17

**Authors:** Nina Ventura, Edson Marchiori, Gláucia Zanetti, Antonio Muccillo, Mariana Leite Pereira, Guilherme Abdalla, Pedro Martins, Carolina Lamas Constantino, Rodrigo Canellas, Viviane Brandão, Romulo Varella de Oliveira

**Affiliations:** Department of Radiology, Rio de Janeiro Federal University, Rio de Janeiro, CEP 21941.913, Brazil

## Abstract

Medullary thyroid carcinoma is a rare malignancy that arises from calcitonin-producing C-cells and frequently metastasizes to lymph nodes in the neck. Distant metastases may involve bone, lung, and liver. The infrequent number of cases limits the clinical nature and ability to optimize diagnostic tools. Here, we present a case of a micronodular radiographic pattern in metastatic medullary thyroid cancer in order to enhance awareness of the disease process. A case discussion and relevant review of the literature are provided.

## 1. Introduction

Medullary thyroid carcinoma (MTC) is an uncommon primary thyroid tumor that accounts for as much as 5%–10% of all thyroid malignancies [1–5] and as much as 13% of all thyroid cancer-related deaths [4, 5]. MTC lesions can metastasize to regional lymph nodes but may also spread hematogenously and affect the liver, bone, and lungs [6]. Metastases to the lung generally exhibit a macronodular appearance [7, 8], but other pulmonary patterns have been described in the literature. The diverse radiographic manifestations of pulmonary MTC metastases pose a diagnostic challenge for clinicians. In this paper, we present the case of a 40-year-old man diagnosed with MTC and micronodular lung metastases.

## 2. Case Report

A 40-year-old man was admitted to the hospital with complaints of diarrhea and an unintentional weight loss of 5 kg over the past 6 months. The patient was in good condition and had a physical examination that was normal, with the exception of thyroid swelling, and an enlarged cervical node that was revealed during examination of the neck. The hemogram, urea, creatinine, liver function tests, and urinalysis were all normal. 

Tests of thyroid function indicated a thyroid-stimulating hormone level of 1.05 uIU/mL (reference range: 0.4–4.0 uIU/mL), thyroglobulin of 16.0 ng/mL (reference range: 5–50 ng/mL), thyroglobulin antibody of 8.0 U/mL (reference range: 1–10 U/mL), free tyrosine of 1.6 ng/dL (reference range: 0.8–1.9 ng/dL), triiodothyronine 118 ng/dL (reference range: 60–181 ng/dL), antiperoxidase of 12.4 U/mL (reference range: under 15 U/mL), and sedimentation rate of 6 mm^3^/hour (reference range: 0–15 mm^3^/hour). 

Thyroid ultrasonography showed a hypoechogenic 3 cm mass in the right lobe of the thyroid. Scintigraphy revealed an enlarged right lobe that showed no concentration of radiotracer. A fine-needle aspiration from the thyroid mass revealed that cells had an outlining glandular arrangement and were consistent with malignant epithelial tumor cells. 

The absence of family history of MTC indicated that the carcinoma present was of a sporadic nature. Pheochromocytoma was excluded based on a normal abdominal computed tomography (CT) scan, normal plasma catecholamine levels, and excretion of metanephrine and normetanephrine in a urine sample collected after 24 hours. The serum preoperative calcitonin level was significantly elevated at 22.450 pg/mL (reference range: 0–42 pg/mL), and carcinoembryogenic antigen (CEA) level was 574 g/mL (reference range 0–3 ng/mL). The patient underwent a total thyroidectomy with bilateral neck dissection. Histopathological examination revealed a thyroid medullary carcinoma with metastasis to cervical lymph nodes in levels II, III, and IV. As a result, surgical staging was considered IVb (T2 N1b M0). Immunohistochemical analyses detected expression of antithyroglobulin, anticalcitonin, and antichromogranin A. Postoperatively, calcitonin levels decreased to 382 pg/ml and CEA levels decreased to 309 ng/mL. An additional thyroid ultrasonography was performed, and a persistent hypoechogenic 2.1 cm mass was detected in the right lobe of the thyroid. Abdominal and thorax CT scans were normal. 

One month after surgery, the patient underwent 25 sessions of radiotherapy of the cervical and upper mediastinum. Within 6 months from the start of the therapy, the levels of CEA and calcitonin slowly decreased to 243 ng/mL and 214 pg/mL, respectively. The patient failed to attend the control visits. 

**Figure 1:**
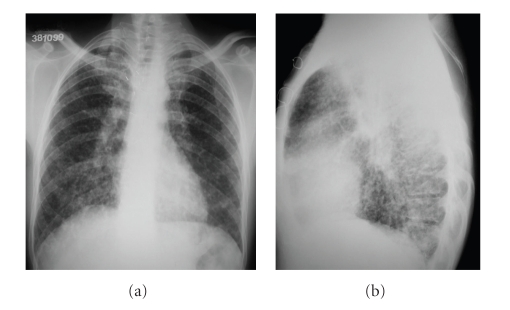
Chest radiographs in anteroposterior (a) and lateral (b) incidences showing infiltration by small nodules disseminated in both lungs.

**Figure 2:**
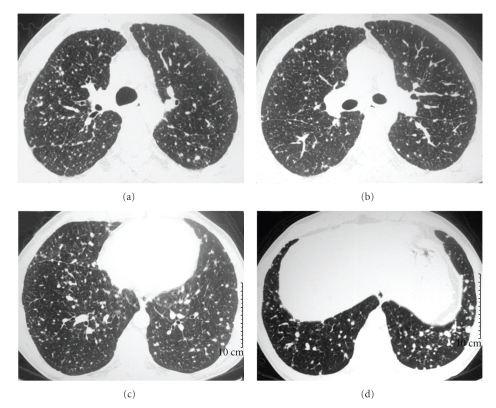
High-resolution CT scans at four different levels showing small nodules distributed randomly through the lungs. Note that the nodules are larger in the lung bases (d).

The patient went to the hospital two years after the treatment with complaints of wasting syndrome and chronic diarrhea (10 episodes/day). The laboratory tests showed calcitonin levels of 32.137 pg/mL and CEA levels of 658.8 ng/mL. A plain chest radiograph showed a diffuse micronodular pattern in both lung fields (Figure 1). A bronchoscopy performed was normal and the bronchoalveolar lavage and culture for mycobacteria and fungi were negative. There was also an absence of neoplastic cells. A CT of the thorax revealed multiple pulmonary nodules that were suggestive of metastases (Figure 2). When an abdominal ultrasound was performed, metastatic disease in the liver was detected that consisted of two hyperechogenic nodules, a 10 mm nodule in segment VII and a 9 mm nodule in segment VIII.

The patient was discharged one month later and was given octreotide (Sandostatin LAR), Puran T4, CaCO3, and vitamin D3 to take for treatment. The decision to start octreotide was based on positive expression of somatostatin receptors detected on an octreoscan. Three months after discharge, the patient is undergoing ambulatory follow-up and has a satisfactory clinical response with weight gain, a reduced incidence of diarrhea, and normal laboratory tests resulting in a significant improvement in the patient's overall quality of life.

## 3. Discussion

Medullary thyroid carcinoma arises from the parafollicular cells or C-cells of the thyroid gland, which produce calcitonin and are neuroendocrine in origin [6]. In MTCs, the calcitonin levels are critical for both diagnosis and postoperative surveillance [6]. MTC can occur as a noninherited, sporadic disease that typically presents in the fourth decade of life. It can also be transmitted genetically in an autosomal dominant inheritance pattern that is associated with either familial medullary carcinoma of the thyroid (FMTC), multiple endocrine neoplasia (MEN)-IIA (MTC, pheochromocytoma, and hyperparathyroidism), or MEN-IIB (MTC, pheochromocytoma, mucosal neuroma, and marfanoid habitus). The sporadic variant is typically unilateral and corresponds to approximately 75% of all MTC cases [6]. 

At the initial diagnosis, the sporadic variant of MTC often presents with distant metastasis involving cervical lymph nodes, lungs, liver, and bones and is the main cause of MTC-related death [4, 9]. Metastases to the brain and skin are not observed as frequently. Distant metastases are usually diffuse and abundant and generally affect multiple organs [1]. Despite a high rate of metastasis to the lymph node, the 5-year and 10-year survival rates for MTC are between 78%–91% and 61%–75%, respectively [6, 10]. In general, the overall survival rate of patients with MTC is intermediate to that of patients with differentiated thyroid carcinoma (papillary and follicular) and anaplastic carcinoma [11, 12]. Patients with the sporadic form of MTC and who have systemic symptoms generally have broad metastatic disease [11]. Of those patients, 33.3% die within 5 years [11]. Our patient presented the sporadic form of the disease.

Prognostic factors that are relevant to the outcome of patients diagnosed with MTC include the age at diagnosis, male gender, the initial extent of the disease (including lymph node and distant metastases), tumor size, extrathyroid invasion, vascular invasion, calcitonin immunoreactivity, and amyloid staining in tumor tissue [1, 11, 13, 14]. A total thyroidectomy with meticulous triple compartment nodal dissection correlates with the highest survival rate for patients. Therefore, aggressive methods to detect and characterize tumor behavior as early as possible are the best approaches for increasing patient survival rates [7, 15]. The benefits of adjuvant external beam irradiation to the neck (EBRT) have been poorly evaluated, remain controversial, and are not predicted to provide normalization of serum levels of calcitonin [16]. However, in high risk patients with microscopic residual disease, extraglandular invasion, or lymph node involvement, the local/region relapse-free rate was found to be 86% after 10 years following treatment with postoperative EBRT versus 52% for patients who did not receive EBRT. EBRT could also be applied to patients with macroscopic disease after incomplete surgery for local disease control [16].

Elevated basal calcitonin concentrations are found in MTC, C cell hyperplasia, and in rare subjects without any C cell abnormalities [1]. The prognostic value of calcitonin immunostaining has been studied in primary [4, 17, 18] and metastatic tumors [4, 16]. These studies concluded that calcitonin-poor primary tumors were associated with lower survival rates [4, 17, 18]. The same was also true for calcitonin-poor metastases [4, 17], which had an aggressive clinical course and poor prognosis [4, 19]. In patients with MTC, the calcitonin levels become undetectable after extensive surgery in most patients without neck lymph node metastases but remain elevated in two thirds of the patients with neck lymph node involvement [1, 20–22]. An elevated calcitonin level indicates persistent disease and requires multiple imaging procedures [19, 23, 24]. CEA is produced by neoplastic C cells. Measurement of serum CEA concentration is useful during follow-up, since high concentrations or rapidly increasing concentrations indicate disease progression [1, 25].

Medullary carcinomas of the thyroid commonly exhibit a macronodular pattern, also known as a “cannonball” appearance, with clear-cut margins [4, 7]. Although the pulmonary micronodular patterns appear more commonly in metastatic papillary thyroid cancer [7, 8], the case presented here indicates that they can also occur in MTC. Unfortunately, a micronodular pulmonary pattern can also be easily mistaken for granulomatous diseases, such as tuberculosis, histoplasmosis, or sarcoidosis, which further complicate the diagnosis [7]. A diagnosis is based on the patient's history, clinical assessment, tumor markers, and histopathological examination of the primary or metastatic lesion. In addition to an MTC diagnosis, an accurate determination of the extent of disease is important for the initial treatment [8, 26]. Reticulonodular perihilar lesions [4, 7, 27] and calcified pulmonary metastases [2, 4, 7, 28] have also been reported in the literature, and it is known that the incidence of calcified metastasis at the initial presentation is high in patients with MTC [8, 26, 29]. In a review of the literature, very few cases have been reported to have micronodular patterns [7, 27]. Metastatic disease is an important cause of micronodular pulmonary patterns. Since metastases are detected after the primary lesion is identified, a presumptive diagnosis of metastasis from the known primary lesion is an accurate diagnosis.

It is also important to consider that there is currently no single, sensitive diagnostic imaging modality for the detection of all metastases present in patients with MTC. Therefore in many cases, several imaging modalities are employed, such as ultrasonography, computerized tomography, scintigraphy using pentavalent technetium-99m dimercaptosuccinic acid, thallium-201 chloride, and indium-111 pentetreotide, as well as fluorine-18 fluorodeoxyglucose positron emission tomography (18F-FDG PET/CT). The consecutive administration of these techniques has been applied to patients with elevated calcitonin levels until tumors present were detected [30]. Thallium 201-chloride single-photon emission CT has also been shown to detect metastatic differentiated thyroid carcinoma foci as small as 1 cm in the neck, 1.5 cm in the lung, and disseminated micronodular pulmonary metastases, especially in patients whose scans were negative following administration of 131I [7, 31]. A short review by Conry et al. [32] showed that neither 18F-FDG PET/CT, nor the novel PET somatostatin analogue, 68Ga-DOTATATE PET/CT, can fully map the extent of disease in patients with recurrent MTC, although 18F-FDG PET/CT may identify more lesions. 68Ga-DOTATATE PET/CT also represents a useful, complementary imaging tool for the detection of the extent of disease and has the potential to identify patients that may qualify for targeted radionuclide somatostatin analogue therapy.

Finally, no effective therapy is currently available for the management of patients with MTC. According to Leboulleux et al. [1], treatment with radioactive iodine is ineffective since C cells do not take up radioiodine. Alternatively, the efficacy of pretargeted radioimmunotherapy (pRAIT) that uses bispecific monoclonal antibodies in combination with an iodine-131- (131I-) labeled bivalent hapten was evaluated by Chatal et al. [33]. pRAIT was found to provide long-term disease stabilization for patients with CEA and a significantly longer survival for high-risk patients that were characterized with short serum calcitonin doubling times (Ct DTs) of less than 2 years compared to high-risk patients that remained untreated. Ct DT and bone-marrow involvement also appear to be prognostic indicators in MTC patients who undergo pRAIT.

## 4. Conclusion

MTC that exhibits a micronodular pulmonary pattern represents a relatively rare presentation of metastatic MTC, since most pulmonary metastases exhibit macronodular patterns. It is imperative to use all available sources to accurately diagnose and cure this type of aggressive tumor and appropriately exclude inflammatory causes. We have presented a case of medullary thyroid carcinoma with micronodular pulmonary metastases to enhance awareness of this uncommon presentation of a rare and aggressive disease.
